# Effect of Phytochemical-Rich Food Intake on Respiratory and Muscle Function in Middle-Aged Patients with COPD: A Cross-Sectional Study

**DOI:** 10.3390/nu16223962

**Published:** 2024-11-20

**Authors:** Alda Ranogajec, Ana Ilić, Snježana Benko Meštrović, Ivana Rumbak

**Affiliations:** 1 Special Hospital for Pulmonary Diseases, Rockefellerova 3, 10000 Zagreb, Croatia; avilogorac@gmail.com (A.R.); snjezanabenkom@gmail.com (S.B.M.); 2 Department of Food Quality Control, Faculty of Food Technology and Biotechnology, University of Zagreb, Pierottijeva 6, 10000 Zagreb, Croatia; ivana.rumbak@pbf.unizg.hr; 3 Physiotherapy Department, University of North, Jurja Križanića 31b, 42000 Varaždin, Croatia

**Keywords:** chronic obstructive pulmonary disease, dietary phytochemical index, diaphragm thickness, FEV_1_, FVC, MIP, peripheral muscle strength, pulmonary rehabilitation

## Abstract

**Background**: It is known that the consumption of single phytochemicals improves respiratory function in chronic obstructive pulmonary disease (COPD) patients. Since phytochemicals have a synergistic effect on health, a more comprehensive analysis is needed. The aim of this study was to estimate the intake of phytochemicals using the dietary phytochemical index (DPI) and assess their association with respiratory function, inspiratory muscle strength and function, and peripheral muscle strength. **Methods**: This study was conducted at the Special Hospital for Pulmonary Diseases in Zagreb (September 2023 to May 2024). The DPI was assessed using three 24 h recalls from 71 COPD patients (66.5 ± 8.4 years; 53.5% men). Anthropometric measurements, respiratory function, inspiratory muscle function and strength, and peripheral muscle strength were assessed during pulmonary rehabilitation following standard protocols. **Results**: Patients were divided into DPI tertiles with mean values of 7.3 ± 3.0, 16.0 ± 3.0, and 32.2 ± 8.8, respectively. After controlling for confounding factors, a significant association was found between DPI tertiles and FEV_1_ (*p*-trend < 0.001), FVC (*p*-trend = 0.002), FEV_1_/FVC (*p*-trend < 0.001), MIP (*p*-trend = 0.012), and MSUE (*p*-trend = 0.002). In addition, an inverse association was found between DPI tertiles and diaphragm thickness during inhalation (*p*-trend = 0.012) and exhalation (*p*-trend = 0.013). **Conclusions**: This study suggests that a higher intake of phytochemicals could be beneficial in dietary interventions for COPD therapy. Future prospective studies are needed to confirm these findings.

## 1. Introduction

By definition, chronic obstructive pulmonary disease (COPD) is a chronic, non-communicable lung disease characterized by airflow limitation and respiratory symptoms such as shortness of breath, cough, expectoration, wheezing, etc. [1]. COPD is a multifactorial systemic disease that can develop from several causes (alpha-1 antitrypsin deficiency, abnormal lung development, severe respiratory infections and childhood asthma, air pollution, occupational exposures, etc.), with the main risk factor being exposure to tobacco (active or passive) [1,2,3]. It is recognized that some of the comorbidities such as heart disease, cancer, poor mental health, musculoskeletal disorders, gastrointestinal disorders, endocrine and metabolic disorders, sleep disorders, malnutrition, and other diseases associated with poor nutrition are associated with COPD [3]. According to estimates, COPD caused 3.3 million deaths in 2019, making it the third most common cause of death worldwide, and the trend is rising [3,4,5].

The most important treatment approach for patients with COPD is pulmonary rehabilitation, which combines pharmacotherapy and supplemental oxygen therapy with exercise training, education, and behavior change [6]. Education may include nutritional therapy and dietary behavior modification due to the high prevalence of nutritional depletion in patients with COPD [7]. Nutritional interventions that focus on regulating body mass and reducing the degree of malnutrition as part of pulmonary rehabilitation may improve COPD outcomes [8,9]. In addition, according to the systematic reviews and meta-analyses, unhealthy eating habits are associated with a higher risk of COPD [10,11,12]. Most studies focus on improving respiratory function and nutritional status by providing certain nutrients such as protein, vitamin E, and vitamin D or taking Oral Nutritional Supplements (ONSs). Since there has been a recent shift to looking at the diet as a whole rather than focusing on a single nutrient, it has been found that patients who followed the Dietary Approaches to Stop Hypertension (DASH) and the Mediterranean diet (MedDiet) had better respiratory function [13,14,15,16]. Both DASH and the MedDiet are characterized by a high intake of whole grains, fruits and vegetables, and plant-based proteins and a lower intake of red meat, processed foods, and salt [17,18]. Indeed, a higher intake of fruit and vegetables is associated with lower risk of COPD [11,19,20,21]. It has been hypothesized that consumption of fruit and vegetables, as a good source of phytochemicals, may reduce the oxidative stress that often occurs in COPD patients [22,23,24,25]. The link between phytochemical intake and respiratory function in patients with COPD is still unclear. However, in the literature several studies have highlighted the benefit of intake of individual phytochemicals to reduce the symptoms of COPD and prevent lung cancer. COPD is an inflammatory disease and, in addition, most COPD patients were smokers or continue to smoke after diagnosis, which can further exacerbate oxidative damage and prolong immune responses [26]. Therefore, phytochemicals can act as antioxidants and reduce the expression of genes that promote the immune system and increase mucus secretion [26,27]. Carotenoids, of which lycopene is one of the most interesting, are among the phytochemicals proposed as a therapy for patients with lung disease. Carotenoids are thought to activate the NRF2/HO-1 signaling pathway, which inhibits transcription factors, namely nuclear factor-kappa B (NF-κB) [27,28,29]. The second type of interesting phytochemicals is flavonoids, whose higher uptake improves FEV_1_. One of the suggested underlying mechanisms is that they reduce oxidative stress and increase PaO_2_ [30,31]. Moreover, the flavonoid resveratrol from wine appears to inhibit inflammatory cytokines and the activation of NF-κB and AP-1, while stimulating GSH synthesis [32,33]. Curcumin is one of the phytochemicals that have been studied in relation to lung disease. Curcumin is thought to inhibit the synthesis of NF-κB, particularly in smokers, and can enhance the effects of glucocorticoids [34,35].

Phytochemicals are secondary metabolites that are mainly found in plant foods and whose intake has a positive effect on health [36,37]. However, there is no recommendation for their intake as there is lack of information on their quantity in foods and their bioavailability and accessibility after processing and metabolism [36,38,39,40]. To address this problem, McCarty proposed the dietary phytochemical index (DPI) as a tool to determine the intake of phytochemicals [41]. The DPI is based on the daily energy intake from foods rich in phytochemicals. Therefore, the DPI enables the indirect assessment of the intake of various phytochemicals through the assessment of food intake. Further, the DPI has been used in several epidemiologic studies and linked phytochemical intake to disease outcomes, e.g., migraine [42], gastric ulcer [43], multiple sclerosis [44], various cancers [45,46,47,48,49], mental disorders [50,51], metabolic syndrome [52], skeletal disorders [53,54], cardiovascular diseases [55,56,57,58], nutritional status [59,60], non-alcoholic fatty liver disease [61], etc. To our knowledge, this has not yet been observed in patients with COPD.

To better understand the relationship between the intake of phytochemicals and the health status of adult COPD patients, this study aims to estimate the intake of phytochemicals using the DPI and to observe the relationship between the DPI and respiratory function (forced expiratory volume in the first second and forced vital capacity). Since muscle strength is a risk factor for poorer outcomes, this study also aims to observe the association between the DPI and inspiratory muscle strength and function (maximum inspiratory pressure, diaphragm thickness in inhalation and exhalation), and peripheral muscle strength.

## 2. Materials and Methods

### 2.1. Settings and Participants

This cross-sectional study was conducted from September 2023 to May 2024 at the Special Hospital for Pulmonary Diseases in Zagreb, Croatia. Participants were recruited via the day clinic during pulmonary rehabilitation. The inclusion criterion was the presence of COPD, which was assessed using the Global Initiative for Chronic Obstructive Lung Diseases (GOLD) scale [62]. During the recruitment phase, patients were recruited who were diagnosed with COPD between 6 and 30 years ago. The exclusion criteria were as follows: use of long-term oxygen therapy; overlapping conditions with asthma; use of systemic corticosteroid therapy in the last six months; implanted pacemaker or other electrical implant; neuromuscular disease, phrenic nerve injury, or a condition following cerebrovascular accident with residual hemiparesis; history of lung surgery; and autoimmune, neurological, or other systemic diseases that could affect respiratory function. During the recruitment period, a total of 102 patients were eligible for the study. However, after applying the exclusion criteria, 73 patients were included in the study (72%). After data collection, two additional patients were excluded for the purposes of the present study because they had not completed the 24 h recalls. Finally, data from 71 (70%) adult patients with COPD (45 to 85 years) were analyzed. An a priori analysis to estimate the sample size was performed using the GPower program (version 3.1.9.2; Heinrich Heine University Dusseldorf, Dusseldorf, Germany). The a priori analysis was performed assuming a power of 80%, a significance level of α = 0.05, an expected effect of 0.15, and at least 2 existing predictors (sex and smoking). Respiratory function was used as the primary outcome to assess the expected effect. This analysis indicated that at least 68 patients should be included in the study. General information about the participants was collected through interviews during pulmonary rehabilitation and through the Hospital Information System software (version 212.0.000¸IN2 Ltd., Zagreb, Croatia).

Each patient was informed of the study protocols and gave written consent to participate in the study. Participants were free to withdraw their consent at any time during or after data collection. The study protocols were designed and conducted in accordance with the Declaration of Helsinki. In addition, the protocols were approved by the Ethics Committee of the School of Medicine, University of Zagreb (reference number: 251-59-10106-23-111/203, class: 641-01/23-02/01).

### 2.2. Dietary Assessment

Data on consumed food and drink were collected using 24 h recalls on three non-consecutive days within the first two weeks of recruitment. Each patient was interviewed individually by trained dietitians from the research team using a 5-step protocol. Portion size was estimated using kitchen utensils (cups, spoons, plates, etc.), packaging/portion sizes of packaged foods, or in grams if participants knew the weight of food and drink consumed. In addition, the brands of packaged foods and food supplements were recorded. The conversion of the collected data into energy and macronutrient intake was carried out using the Prehrana software (version 1.0; Infosistem Plc., Zagreb, Croatia). The software contains national food composition tables [63] and is supplemented by data on packaged foods and food supplements from their nutrition labels. The observed individual means method was used to present daily energy and energy intake from food items that are considered good sources of phytochemicals [64].

### 2.3. Dietary Phytochemical Index

For each patient, the DPI was calculated according to the proposed method as the ratio between the daily energy intake from foods that are good sources of phytochemicals and the total daily energy intake multiplied by 100 [41]. Food items that are high in phytochemicals include the following: fruit, including natural fruit juices, vegetables (including natural vegetable juices and tomato sauces), plant-based protein food (dry legumes, nuts, and seeds), whole grains (grains, flour/semolina, bread and rolls, cereals, and pasta), and other phytochemical-rich food (herbs, spices, coffee, tea, soy products, cocoa, beer and wine, and olive oil). Due to their low content of phytochemicals, potatoes and picked fruit and vegetables were excluded from the estimation of the DPI [41,46,53,58,59].

### 2.4. Anthropometric and Body Composition Assessments

An anthropometric assessment was performed during the visit to the pulmonary rehabilitation day clinic. Body height was measured to the nearest 0.1 cm using a stadiometer (SECA 799, Hamburg, Germany). Body weight was measured to the nearest 0.1 kg using a combination digital scale with bioelectrical impedance (TANITA MC-780MA P, Tanita Corp., Tokyo, Japan). Body mass index (BMI; kg/m^2^) was calculated for each patient based on body mass and weight measurements. Patients were classified according to the BMI classification proposed by the World Health Organization [65].

The body composition assessment (TANITA MC-780MA P, Tanita Corp., Tokyo, Japan) includes several analyses from which the percentage of body fat and appendicular muscle mass (kg) were extracted for the purposes of this study. The appendicular skeletal muscle index (SMI; km/m^2^) was calculated for each patient from the appendicular muscle mass data obtained. The cut-off value for men and women for the SMI was taken from the ESPEN guidelines [66].

### 2.5. Respiratory Function Assessments

FEV_1_ and FVC were measured with a Medisoft HypAir PFT System (Medisoft, Sor-rines, Belgium) in seated position. Expiratory flow is measured by having the patient inhale as deeply as possible and exhale forcefully and completely as quickly as possible into a device that records the volume of exhaled air (FVC) as a percentage of the predicted value for age and sex and the volume of air exhaled in the first second (FEV_1_) as a percentage of the predicted value for age and sex. It is normally 80% of the FVC. We repeated the measurement at least 3 times with an interval of 90 s between each measurement in COPD patients and recorded the highest value obtained [67].

### 2.6. Inspiratory Muscle Strength and Function Assessment

The assessment of inspiratory muscle strength and function includes maximum inspiratory pressure (MIP) and diaphragm thickness at the end of inhalation (T min) and exhalation (T max).

MIP was measured using the PowerBreathe device (PowerBreathe International Ltd., Southam, England, UK). The MIP value reflects the strength of the diaphragm and the extra-diaphragmatic inspiratory muscles. The patient should be in a sitting position and have a blocked nose, for which a tick is provided. The patient first inhales and exhales normally through the mouthpiece several times, then exhales slowly and to the end (to the residual volume), and then inhales as forcefully as possible. The breath must last at least 1.5 s and the maneuver is repeated five times. The highest value achieved is recorded, not the mean value (Sachs et al., 2009). The device calculates the expected MIP value based on the age and sex entered using the formula for males 120 − (0.41 × age) and for females 108 − (0.61 × age). Values <60 cmH_2_O in adults under 40 years, <50 cmH_2_O in adults between 40 and 80 years, and <40 cmH_2_O in adults over 80 years indicate weakness of the of the diaphragm and extra-diaphragmatic inspiratory muscles [68,69].

The measurement of diaphragm thickness (mm) at the end of inspiration and expiration was performed with the ultrasound device Mindray DC-8 (Mindray Medical International Ltd., Shenzhen, Guandgong, China), with a linear probe frequency of 7.5–10.0 MHz with the patient in supine position in B-mode. The probe was placed above the apposition zone, between the eighth and ninth intercostal spaces, 0.5–2.0 cm below the costophrenic angle, between the anterior and middle axillary line on the right side. The thickness of the diaphragm was measured at the end of inspiration and at the end of expiration. The measurements are presented as a three-layered structure consisting of a hypoechoic inner muscle layer surrounded by two hyperechoic outer membranes (peritoneum and pleura). To objectively quantify the thickening of the diaphragm, at least three measurements are required and the data are presented as an average of the measurements. Here, 2.1 ± 0.4 (1.3–3) mm in men and 1.9 ± 0.4 (1.1–2.7) in women are cut-off values for diaphragm thickness (mm) at the end of inspiration and 2.8 ± 0.6 (1.7–3.9) in men and 2.5 ± 0.6 (1.3–3.7) in women for diaphragm thickness at the end of expiration [70,71].

### 2.7. Peripheral Muscle Strength Assessment

The peripheral muscle strength assessment includes the strength of forearm flexors (*m. biceps brachii*; MSUE; N) and of the leg extensors (*m. quadriceps*; MSLE; N). Both were measured using the digital dynamometer Pelican 1150 (Lafayette Instrument Company, Lafayette, LA, USA) in accordance with standard protocols [72]. The strength of the biceps brachii muscle was measured in a sitting position with the arm bent at the elbow by 90 degrees. The researcher placed the dynamometer on the distal part of the forearm and the patient flexed the arm at the elbow against the resistance of the researcher. The strength of the quadriceps muscle was measured in a sitting position with the knee bent at 90 degrees. The researcher placed the dynamometer on the distal part of the lower leg and the patient stretched their leg against the resistance of the researcher. The measurement was repeated three times and the mean value was recorded.

### 2.8. Statistical Analysis

All statistical analyses were performed using SPSS software (IBM SPSS Statistics for Windows, version 23.0. Armonk, NY, USA: IBM Corp.) with a significance level of α = 0.05. The normality of the distribution of the data was checked using the Shapiro–Wilk test. Only seven variables had missing data (less than 5%), for which we performed a series of mean imputations. Patients were classified into tertiles of the DPI score using the visual binning method. Accordingly, 23 patients were classified into the first tertile of the DPI (DPI ≤ 11.2) and 24 patients in the second (DPI 11.3–20.4) and third (DPI ≥ 20.5). General characteristics, anthropometric measurements, body composition, and dietary habits were presented as the mean and standard deviation for continuous variables and as a percentage for categorical variables according to the tertiles of the DPI. Comparisons between tertiles were performed using a one-way analysis of variance (ANOVA) with a post hoc Bonferroni test for continuous variables and a chi-square test or Fisher’s exact test for categorical variables. Associations between continuous dependent variables (upper and lower muscle strength, diaphragm thickness ate the end of inhalation and exhalation, MIP, FEV_1_, FVC, FEV_1_/FVC) and the tertiles of the DPI were observed using multivariate linear regression in crude and adjusted models. In the first model, we controlled for sex (categorical), age (continuous), number of diagnoses (continuous), GOLD stage (categorical), smoking (categorical), and daily energy intake (continuous). In the second model, we additionally controlled for body mass index (continuous), percentage of body fat (continuous), and the skeletal muscle index (continuous).

## 3. Results

A total of 71 patients with COPD with an average age of 66.5 ± 8.4 years took part in this study. The study population was almost equally divided between men (53.5%) and women (46.5%). According to the GOLD diagnostic protocol, most patients had GOLD stage 2 COPD. In addition to COPD, 30% of patients had one to three other dual diagnoses, of which hypertension (36.6%), emphysema (12.7%), and dyslipidemia (11.3%) were the most common. In addition, more than half of the patients were former smokers.

**Figure 1:**
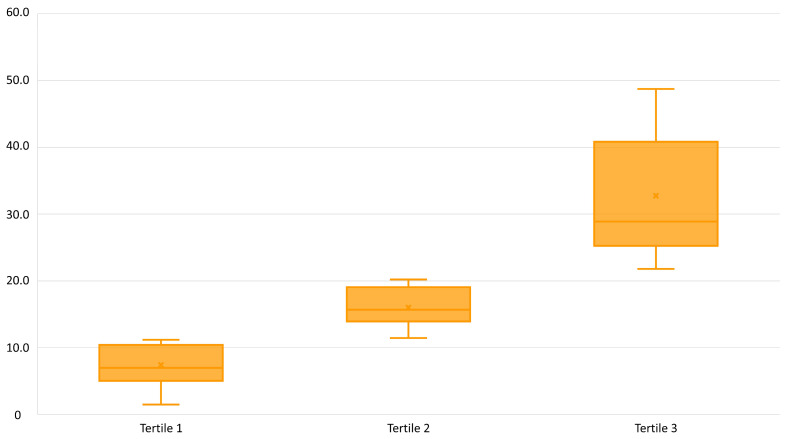
Descriptive statistics of the dietary phytochemical index across tertiles of patients with COPD (*n* = 71).

**Figure 2:**
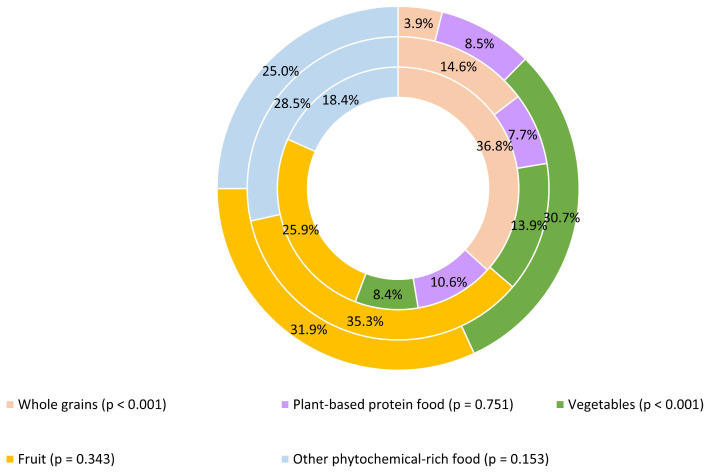
Relative contribution of the food groups to the daily DPI across the DPI tertiles. The external circle represents the first tertile, while the inner circles represent the next higher tertile. The differences between the tertiles of the DPI were tested using a one-way analysis of variance with the post hoc Bonferroni test (*p* < 0.05).

The average DPI in this study population was 18.7 ± 11.8. For further analysis, the study population was divided into tertiles according to the DPI. The descriptive statistics of the DPI for each tertile are shown in Figure 1. The average DPI of the patients in the first tertile was 7.3 ± 3.0, in the second 16.0 ± 3.0, and in the third 32.2 ± 8.8. The average contribution of the food groups to the DPI is shown in Figure 2. The foods from the whole grain group had the lowest contribution to the DPI in the first tertile and the contribution increased significantly (*p* < 0.001) until the last tertile. Conversely, the foods from the vegetable group contributed the most to the patients’ DPI in the first tertile, with the contribution decreasing significantly (*p* < 0.001) by the third tertile.

The differences in the general characteristics of the study population between the tertiles of the DPI are shown in Table 1. The only significant difference (*p* = 0.044) was observed between sexes, with more males (73.9%) in the first tertile of the DPI and more females (62.5%) in the third tertile of the DPI.

Assessment of anthropometry and body composition revealed that the majority of patients had a normal weight status (43.7%), with an average body fat percentage of 27.5% ± 9.1%. According to the skeletal muscle index, the majority of patients (83.1%) had normal appendicular muscle mass. No differences were found in the anthropometric measurements and body composition of the patients across the tertiles of the DPI (Table 2).

The dietary habits of patients with COPD according to the tertiles of the DPI are shown in Table 3. On average, patients had a daily energy intake of 1529 ± 405 kcal, with patients in the lowest and highest tertiles having a significantly lower (*p* = 0.034) energy intake than patients in the second tertile. The proportion of proteins, carbohydrates, and fats in the daily energy intake did not differ between the patients across the DPI tertiles.

The association between respiratory function and muscle strength with the DPI is shown in Table 4. No associations were found in unadjusted analyses. After adjustment for sex, age, number of diagnoses, smoking, GOLD stage, and daily energy intake, multivariate linear regression reveals that the DPI is associated with FEV_1_ (*p*-trend < 0.001), FVC (*p*-trend = 0.001), FEV_1_/FVC (*p*-trend < 0.001), and MSUE (*p*-trend < 0.001). Furthermore, after additional adjustment for body mass index, percent body fat, and the skeletal muscle index, the association between the DPI and FEV1 (*p*-trend < 0.001), FVC (*p*-trend = 0.002), FEV_1_/FVC (*p*-trend < 0.001), and MSUE (*p*-trend = 0.002) remained unchanged, while a positive association between the DPI and MIP (*p*-trend = 0.026) and an inverse association between diaphragm thickness at the end of inhalation (*p*-trend = 0.012) and end of exhalation (*p*-trend = 0.013) emerged.

## 4. Discussion

To our knowledge, this cross-sectional study is the first to observe an association between respiratory function and muscle strength and comprehensive intake of phytochemicals, estimated by the DPI, in patients with COPD. In terms of respiratory function, we observed a relationship between FEV_1_, FVC, FEV_1_/FVC, MIP, and the DPI and an inverse relationship between diaphragm thickness during inhalation and exhalation. We also observed a positive relationship between upper muscle strength and the DPI. Associations were significant only after controlling for covariates.

COPD is one of the chronic diseases with a high mortality rate, whose prevalence has been increasing for decades [3,4,5]. The different outcomes of COPD, such as emphysema, chronic bronchitis, asthma, cough, expectoration, wheezing, etc., can significantly affect the patient’s quality of life [73,74]. Malnutrition is one of the most important comorbidities in patients with COPD, which can worsen the above-mentioned consequences and the quality of life [2,75,76]. Indeed, nutritional support to treat malnutrition in patients with COPD can improve quality of life [77,78,79]. Moreover, the dietary patterns that are described as healthy ones and imply consumption of whole grains, fish, fruit, and vegetables may lower the risk of COPD [10,80,81,82]. The existing results on the effect of dietary patterns on respiratory capacity are inconclusive. Several prospective studies have shown that healthy dietary patterns can increase FEV_1_ and FVC [81,83,84]. On the other hand, Ardestani et al. [14] found that COPD patients adhered to the DASH diet less well than the control group and that there is no correlation between adherence to the DASH diet and FEV_1_ or FVC. The possible explanations for the potential benefits of this diet are seen in the higher intake of vitamins and phytochemicals with antioxidant potential as well as in the intake of dietary fiber. Patients with COPD have higher levels of oxidative stress compared to healthy people [85,86]. The published literature indicates that vitamin C, vitamin E, vitamin A, carotenoids, and phenols may have a positive effect on COPD and respiratory capacity [25,87,88].

To date, over 100,000 different phytochemicals have been identified in food and drinks [36]. Daily intake of phytochemicals cannot be observed in a comprehensive way due to the lack of data on their quantity in food and drinks. However, it has been shown that a variety of phytochemicals in a complex mixture, such as in food, have a stronger antioxidant effect than the intake of a single phytochemical. Therefore, we chose the DPI as an indirect measure of the intake of phytochemicals through the consumption of phytochemical-rich food. Namely, the DPI is calculated as percentages of energy intake from the phytochemical-rich food in daily energy intake. The phytochemical-rich food includes fruit, vegetables, whole grains, legumes, seeds, nuts, olives, olive oil, herbs, spices, coffee, tea, soy products, cocoa, beer, and wine [41]. In line with the results of previous studies on the benefits of consuming certain phytochemicals in people with COPD [25,27], we hypothesized that the synergistic effect of phytochemicals would affect respiratory function. Therefore, we hypothesized that patients with a higher DPI would have better respiratory function (FVC, FEV_1_, and FEV_1_/FVC), which was confirmed by the results of our study. Although we do not know the diversity of phytochemical content in the patients’ diets, these results suggest that dietary patterns consisting of the consumption of foods high in phytochemicals may have a positive impact on COPD outcomes. Therefore, promoting the consumption of phytochemical-rich food could be one of the educational nutritional strategies in COPD rehabilitation programs. However, future randomized controlled studies with a precise dietary plan on the amount of the various phytochemicals are required to determine consumption recommendations.

Measurements of FEV_1_, FVC, and their ratio are the gold standard for the diagnosis of COPD [62]; however, we also measured inspiratory muscle strength and function with the MIP and diaphragm thickness at the end of inhalation (T min) and end of exhalation (T max). These respiratory muscles are important for alveolar ventilation and their impairment can worsen COPD outcomes in patients [89,90]. The pathophysiological process of COPD can lead to a shortening of the diaphragm by reducing the fiber-contracting protein and promoting oxidative stress [91,92]. To our knowledge, this study is the first to examine inspiratory muscle strength and function in relation to dietary habits. We hypothesized that patients with a higher DPI would have a larger layer of diaphragm due to the antioxidant capacity of the phytochemicals. In addition, antioxidants could reduce fatigue during exercise and promote the muscle fiber adaptation process in athletes [93]. Therefore, we hypothesized that higher consumption of foods high in phytochemicals may be associated with better MIP measurements. The results show that the DPI was inversely associated with diaphragm thickness at the end of inhalation and exhalation after adjustment for sex, smoking, GOLD stage, number of diagnoses, daily energy intake, body mass index, percent body fat, and the skeletal muscle index. Diaphragm thickness was lower in patients in the third tertile than in the second tertile of the DPI. Since there is no available literature on the relationship between dietary factors and respiratory muscle function and strength, we cannot assume that only the consumption of phytochemicals causes these findings. However, it seems interesting to point out that patients in the second tertile had, on average, a higher energy intake than patients in the other tertiles, and the average energy intake of patients in the second tertile was in line with the European Food Safety Authority recommendation for energy for adults aged 60-69 years with a physical activity level of 1.4 [94]. On this point, we recommend that future studies focus on this topic. Meanwhile, the present study showed a positive relationship between the DPI and MIP after adjustment for covariates. This suggests that increased consumption of foods high in phytochemicals may have a positive effect on respiratory muscle strength. Inspiratory muscle strength and function is generally higher in men and non-smokers, while it declines with age [95]. In addition, in the available literature malnutrition is associated with lower inspiratory muscle strength and function [90,96,97,98]. In the present study, the age and presence of smokers are similar across the tertile, while there are significantly more men in the first tertile and significantly more women in the third tertiles. The majority of patients in our study had an adequate body mass index and skeletal muscle index.

The results of the existing literature linked the strength of the inspiratory and peripheral muscles in patients with COPD [99]. Malnutrition and reduction in muscle mass is common in patients with COPD [1,2,4]. Two recent randomized clinical trials have shown that protein supplementation can increase peripheral muscle strength in patients with COPD [100,101]. However, to our knowledge, no other dietary component or dietary pattern has been observed in relation to peripheral muscle strength in patients with COPD. In terms of the peripheral muscle strength, we hypothesized that a higher intake of phytochemical-rich food could reduce the oxidative stress, consequently reducing muscle fatigue and damage to the skeletal muscle [93,102]. Accordingly, we examined the relationship between the DPI and MSUE and MSLE, where a positive relationship emerged only for MSUE and was higher in the second tertile than in the third tertile after controlling for the covariates in the analysis. One possible explanation for the extent of the correlation could lie in the higher energy intake of the patients in the second tertiles. However, since we did not observe a direct correlation, we cannot draw any conclusions. In addition, in the present study, we found no difference between the tertiles of the DPI with respect to the contribution of protein to energy intake.

To our knowledge, this is the first study to estimate the DPI in patients with COPD, with an average value of 18.7 ± 11.8 in the total study population (≤11.2 DPI in the first tertile, 11.3–20.4 DPI in the second and ≥20.5 DPI in the third). Furthermore, the DPI has not been estimated in the healthy adult population in Croatia, nor in the adult population with chronic non-communicable diseases, but only in school-age children, in whom the median DPI was 11.8 (7.7–16.2) [103]. However, the DPI has been used in cross-sectional and case–control studies and has been associated with health outcomes in several chronic non-communicable diseases. In the last five years, for example, it has been associated with outcomes, disease severity, or the odds ratio of [42] various cancers [45,46,47,48,49], metabolic syndrome [52], skeletal disorders [53,54], cardiovascular diseases [56,57,58], nutritional status [59,60], and non-alcoholic fatty liver disease [61]. Younger adults (mean age 36.1 years) with migraine headaches had a DPI of up to 17.99 in the first tertile, 17.99–25.93 in the second tertile, and more than 25.93 in the third tertile [42]. Men aged 57.17 ± 8.04 years with benign prostatic hyperplasia had an average DPI of 25 ± 11 and men aged 65.5 ± 13.0 years with prostate cancer had a DPI of 20.4 ± 9.9, which was significantly lower in both studies compared to the healthy control group [45,46]. Patients with colorectal cancer (58.2 ± 10.4 years) had a mean DPI of 19.8 ± 16.0 [47], while the DPI in patients with glioma (aged 20 to 75 years) ranged from 21 (lowest tertile) to 30 (highest tertile) [48]. The highest DPIs were observed in women with breast cancer (62.4 ± 10.8 years), with an average DPI of 54.76 ± 12.96 [49]. In all studies, the cancer was newly diagnosed or no more than 6 months old, and a lower cancer rate was associated with a higher DPI. With regard to skeletal diseases, premenopausal (DPI range from 7.45 to 23.33 by quartile) and postmenopausal (DPI range from 8.00 to 22.04 by quartile) women with a higher DPI had a lower risk of osteoporosis [53], and patients (49.2 ± 8.2 years) with knee osteoarthritis (mean DPI 28.5 ± 7.2) had a lower DPI than the control group [54]. In addition, patients in the highest quartiles/tertiles of the DPI had a lower risk of metabolic syndrome (age 20–75 years; mean DPI 23.13 ± 9.95) and stroke (mean age 64.8 years; DPI range by tertiles from 21 to 29) [52,56]. In terms of nutritional status, the participant (44.7 ± 10.8 years; mean DPI 26.23 ± 9.48) who manifested a metabolically unhealthy overweight/obesity phenotype had a lower DPI [59]. However, general obesity was not associated with the DPI in men (33.7 ± 24.7) and women (36.2 ± 26.8), and only some of the indicators such as waist circumference in women and waist-to-hip ratio in men were associated with the DPI [60]. The study in which a lower DPI was observed concerned patients with non-alcoholic fatty liver disease. Here, the DPI was 17.4 ± 8.7, but the odds ratio decreased significantly across the DPI tertiles [61]. In our study, the observed DPI was 5 or more units less than in the aforementioned studies. This difference could influence the direction and strength of the relationship between the DPI and respiratory function, inspiratory muscle strength and function, or peripheral muscle strength in the present study. In addition, our patients were not newly diagnosed with COPD, but had a history of 6 to 30 years. It must be emphasized that studies on the DPI in patients with chronic non-communicable diseases have used different dietary methods (FFQ, dietary records, or 24 h recalls), which may influence the estimation of the DPI.

If we look at the contribution of the different phytochemical-rich food groups to the DPI, we can see that the whole grain group contributes 10 times less to the DPI in the first tertile than in the third tertile, which is the opposite for the vegetable group. The food groups that contributed most to the DPI in the first tertile were fruit, vegetables, and other phytochemical-rich foods; in the second tertile, fruit, other phytochemical-rich foods, and whole grains; and in the third tertile, whole grains, fruit, and other phytochemical-rich foods. The phytochemical-rich food group in our study consisted of herbs, spices, coffee, tea, soy products, cocoa, beer and wine, and olive oil. According to the available literature, only one study in the last five years has shown the contribution of food group to the DPI [104]. In younger adults (18–35 years) with diabetes mellitus type 1, fruit contributed over 50% and vegetables over 20% to the DPI, but the authors did not present differences in the contribution of food groups between tertiles of patients. In this study, an association was found between the DPI and a lower risk of hypoglycemia and low high-density lipoprotein cholesterol, but not with other cardiometabolic risk factors [104]. The other studies in which the results of differences in food group consumption between tertiles or quartiles of the DPI were presented as daily energy intake (kcal/day), contribution to daily energy intake (% kcal/day), or amount (g/day). It is therefore difficult to identify similarities and differences between the populations studied. However, it must be emphasized that fruit and vegetable intake increases across tertiles/quartiles along with whole grain intake, except in the study of women with breast cancer, where fruit and vegetable intake was similar across quartiles [42,45,47,48,49,54,56,58,60,61]. We hypothesized that patients in higher tertiles of the DPI would have a higher intake of fruits, vegetables, and whole grains, which could impact better health outcomes in COPD. The hypothesis was based on the knowledge that whole grains are a good source of fiber, and it has been found that people who have higher intake of fiber per day have a lower risk of COPD [80,82,105]. However, the present study did not examine the total fiber intake, but only the energy from whole grains and their products. Furthermore, the present study did not examine the combined fruit and vegetable intake in grams per day. Fruit and vegetable intake was one variable of the DPI, and its contribution to the DPI was presented separately. A higher intake of fruit and vegetables may be beneficial for COPD patients, but the effects of their independent consumption on COPD risk or outcomes are inconsistent [80,83,105,106,107]. In addition, certain fruits and vegetables appear to have a different impact on COPD outcomes [21,108]. It must also be emphasized that we did not observe the consumption of other food groups such as cured or processed meat or fish, which could alter the effects on COPD outcomes [11,82,109,110]. Therefore, future work should take a broader dimension and, in addition to the intake of foods that contribute to the intake of phytochemicals, also investigate other nutritional parameters to assess the full contribution of these foods.

When interpreting the results, we must be aware that this is a cross-sectional study with limited ability to observe a causal relationship between the DPI and COPD outcomes. The sample size and pattern may influence the results of this study. Even though we had a sample that allows the observation of the association between the DPI and COPD outcomes, the sample is small and consists mainly of middle-aged men. The present study population was not newly diagnosed patients with COPD, but patients who have been suffering from COPD for 6 to 30 years. In addition, the majority of our patients had an adequate BMI and a normal SMI. Moreover, the energy intake of the participants in our sample was low, whereas energy intake and energy intake from photochemical-rich foods are the main variables of the DPI. Energy intake was observed using 24 h recalls, which may be biased by the cognitive abilities of elderly patients. To compensate for the potential bias due to daily variation in food intake, we collected three 24 h recalls on non-consecutive days [111]. In addition, the patients did not know exactly on which day they would be interviewed for the 24 h recalls in order to minimize the change in their daily routine and eating habits. However, we did not account for potential seasonal variation. One of the major strengths of the present study was the assessment of objective measures using spirometry, which is the gold standard of the COPD diagnostic protocol [62]. Also, except for respiratory function, we objectively measured the strength and function of inspiratory muscles and the strength of the peripheral muscles, which are important factors in the progression of COPD and mortality [89,90].

## 5. Conclusions

In conclusion, we found a relation between respiratory function, inspiratory muscle strength and function, and peripheral muscle strength with the intake of phytochemicals observed with the dietary phytochemical index. However, our results suggest that phytochemical intake may be inversely associated with diaphragm thickness. It is desirable that future research include a prospective cohort study and controlled clinical trials to investigate the long-term effects of phytochemical intake in patients with COPD and to determine the benefits of increased intake of foods rich in phytochemicals for nutritional therapy in these patients. However, the insights of this study support the promotion of the consumption of foods high in phytochemicals in a nutritional education program as part of COPD rehabilitation.

## Figures and Tables

**Table 1:** General characteristics of patients with chronic obstructive pulmonary disease across the tertiles of the dietary phytochemical index ^1^.

Variables	Total (*n* = 71)	DPI Tertiles			*p*-Values ^2^
		T1 (DPI ≤ 11.2)	T2 (DPI 11.3–20.4)	T3 (DPI ≥ 20.5)	
Age (yr.)	66.5 ± 8.4	68.3 ± 8.6	65.9 ± 7.5	65.5 ± 9.0	0.461
Sex (%)					
Male	53.5	73.9	50.0	37.5	0.044
Female	46.5	26.1	50.0	62.5	
GOLD stage (%)					
1	23.9	17.4	29.2	25.0	0.835
2	42.3	39.1	41.7	45.8	
3	23.9	26.1	25.0	20.8	
4	9.9	17.4	4.2	8.3	
Number of diagnoses (%)					
1	30.0	26.1	21.7	41.7	0.111
2	30.0	30.4	34.8	25.0	
3	17.1	4.3	30.4	16.7	
4	20.0	34.8	8.7	16.7	
5	1.4	4.3	0.0	0.0	
6	1.4	0.0	4.3	0.0.	
Smoking (%)					
No	1.4	0.0	0.0	1.4	0.869
Yes	42.3	39.1	41.7	42.3	
Former	56.3	60.9	58.3	56.3	

DPI—dietary phytochemical index; T—tertile; ^1^ data are shown as mean ± standard deviation or percentage, depending on the type of variable. ^2^ Differences between tertiles of the DPI were tested using one-way analysis of variance with the post hoc Bonferroni test for continuous variables and chi-square test/Fisher’s exact test for categorical variables (*p* < 0.05).

**Table 2:** Anthropometric measurements and body composition of patients with chronic obstructive pulmonary disease across the tertiles of the dietary phytochemical index ^1^.

Variables	Total (*n* = 71)	DPI Tertiles			*p*-Values ^2^
		T1 (DPI ≤ 11.2)	T2 (DPI 11.3–20.4)	T3 (DPI ≥ 20.5)	
Weight (kg)	76.2 ± 17.5	77.6 ± 15.7	78.0 ± 21.6	73.0 ± 14.5	0.552
Height (cm)	171.4 ± 7.9	173.5 ± 7.9	17.8 ± 7.0	168.9 ± 8.3	0.134
BMI (kg/m^2^)	25.8 ± 5.2	25.8 ± 4.9	26.2 ± 6.4	25.5 ± 4.3	0.900
BMI category (%)					
Underweight	7.0	13.0	8.3	0.0	0.295
Normal	43.7	34.8	33.3	62.5	
Overweight	19.7	17.4	25.0	16.7	
Obesity	29.6	34.8	33.3	20.8	
Fat-free mass (kg)	54.4 ± 11.0	57.0 ± 10.5	55.0 ± 12.0	51.5 ± 10.1	0.225
Body fat (%)	27.5 ± 9.1	25.9 ± 9.1	27.8 ± 9.6	28.9 ± 8.8	0.532
SMI (kg/m^2^)	7.4 ± 1.3	7.6 ± 1.3	7.7 ± 1.4	7.1 ± 1.0	0.224
SMI category					
Reduced	16.9	30.4	12.5	16.9	0.152
Normal	83.1	69.6	87.5	83.1	

DPI—dietary phytochemical index; T—tertile; BMI—body mass index; SMI—skeletal muscle index; ^1^ data are shown as mean ± standard deviation or percentage, depending on the type of variable. ^2^ Differences between tertiles of the DPI were tested using one-way analysis of variance with the post hoc Bonferroni test for continuous variables and chi-square test/Fisher’s exact test for categorical variables (*p* < 0.05).

**Table 3:** Dietary habits of patients with chronic obstructive pulmonary disease across the tertiles of the dietary phytochemical index ^1^.

Variables	Total (*n* = 71)	DPI Tertiles			*p*-Values ^2^
		T1 (DPI ≤ 11.2)	T2 (DPI 11.3–20.4)	T3 (DPI ≥ 20.5)	
Energy (kcal)	1529 ± 405	1457 ± 359 ^a^	1701 ± 417 ^b^	1425 ± 393 ^a^	0.034
Protein (% kcal)	16.2 ± 3.0	15.5 ± 2.3	15.7 ± 2.8	17.2 ± 3.5	0.087
Carbohydrate (% kcal)	41.2 ± 7.9	39.1 ± 6.2	42.2 ± 9.7	42.2 ± 7.1	0.289
Total fat (% kcal)	43.1 ± 6.9	45.5 ± 6.5	42.4 ± 7.6	41.5 ± 6.0	0.113

DPI—dietary phytochemical index; T—tertile; ^1^ data are shown as mean ± standard deviation or percentage, depending on the type of variable. ^2^ Differences between tertiles of the DPI were tested using one-way analysis of variance with the post hoc Bonferroni test (differences are indicated with a and b) for continuous variables (*p* < 0.05).

**Table 4:** Multivariate linear regression of the association between muscle strength and respiratory function of patients with chronic obstructive pulmonary disease with the dietary phytochemical index ^1^.

Variables	DPI Tertiles			*p*-Trend
	T1 (DPI ≤ 11.2)	T2 (DPI 11.3–20.4)	T3 (DPI ≥ 20.5)	
FEV_1_ (%)				
Crude ^2^		11.07 (−1.52–23.66)	10.40 (−2.19–23.00)	0.150
Model 1 ^3^	Reference	2.97 (−3.62–9.56)	4.02 (−2.29–10.32)	<0.001
Model 2 ^4^		3.70 (−3.19–10.77)	4.35 (−2.12–10.82)	<0.001
FVC (%)				
Crude		8.08 (−2.83–18.98)	6.11 (−4.93–17.16)	0.309
Model 1	Reference	3.27 (−6.13–12.68)	5.76 (−3.24–14.77)	0.001
Model 2		5.78 (−3.97–15.52)	6.84 (−2.19–15.87)	0.002
FEV_1_/FVC				
Crude		0.06 (−0.04–0.16)	0.06 (−0.04–0.16)	0.406
Model 1	Reference	0.01 (−0.06–0.08)	0.001 (−0.07–0.07)	<0.001
Model 2		0.004 (−0.07–0.08)	−0.002 (−0.07–0.07)	<0.001
MIP (cmH_2_O)				
Crude		3.21 (−9.22–15.65)	1.25 (−11.18–13.69)	0.873
Model 1	Reference	1.39 (−11.65–14.44)	4.69 (−7.79–17.19)	0.056
Model 2		−1.09 (−14.25–12.06)	3.97 (−8.23–16.16)	0.026
Diaphragm thickness—inhalation (mm)				
Crude		−0.20 (−0.54–0.15)	−0.32 (−0.66–0.02)	0.172
Model 1	Reference	−0.19 (−0.44–0.26)	−0.13 (−0.47–0.20)	0.053
Model 2		−0.15 (−0.494–0.19)	−0.12 (−0.44–0.20)	0.012
Diaphragm thickness—exhalation (mm)				
Crude		−0.12 (−0.40–0.17)	−0.27 (−0.55–0.02)	0.182
Model 1	Reference	0.01 (−0.29–0.31)	−0.11 (−0.40–0.18)	0.061
Model 2		−0.09 (−0.39–0.21)	−0.13 (−0.40–0.15)	0.013
MSUE (N)				
Crude		29.80 (−2.67–62.27)	6.81 (−25.32–38.94)	0.165
Model 1	Reference	43.48 (13.67–73.29)	22.59 (−5.95–51.13)	<0.001
Model 2		42.92 (11.49–74.34)	23.66 (−5.48–52.80)	0.002
MSLE (N)				
Crude		8.08 (−20.93–37.09)	14.33 (−14.68–43.34)	0.616
Model 1	Reference	9.50 (−22.44–41.45)	20.51 (−10.07–51.09)	0.252
Model 2		12.61 (−21.08–46.29)	22.29 (−8.65–53.84)	0.387

DPI—dietary phytochemical index; T—tertile; MIP—maximum inspiratory pressure; FEV_1_—forced expiratory volume in the first second; FVC—forced vital capacity; MSUE—upper muscle strength; MSLE—lower muscle strength; ^1^ data are shown as unstandardized β coefficients and the 95% confidence interval. ^2^ Crude values of multivariate linear regression with the DPI as the independent variable (*p* < 0.05). ^3^ Model 1: adjusted for sex, age, number of diagnoses, smoking, GOLD stage, and daily energy intake. ^4^ Model 2: adjusted for sex, age, number of diagnoses, smoking, daily energy intake, body mass index, percent body fat, and the skeletal muscle index.

## Data Availability

The data presented in this study are available on request from the corresponding author, as they are required for the further analysis of the dissertation.
